# Influence of adverse effects of neoadjuvant chemoradiotherapy on the prognosis of patients with early-stage esophageal cancer (cT1b-cT2N0M0) based on the SEER database

**DOI:** 10.3389/fsurg.2023.1131385

**Published:** 2023-04-17

**Authors:** Xiying Cao, Bingqun Wu, Hui Li, Jianxian Xiong

**Affiliations:** ^1^ Department of Thoracic Surgery, The First Affiliated Hospital of Gannan Medical University, Ganzhou, China; ^2^ Department of Thoracic Surgery, Beijing Chao-Yang Hospital, Capital Medical University, Beijing, China; ^3^ Department of Thoracic Surgery, Huaxin Hospital, First Hospital of Tsinghua University Beijing, Beijing, China

**Keywords:** nomogram, esophageal cancer, esophagectomy, survival, SEER database

## Abstract

**Objective:**

To analyze the prognostic impact of neoadjuvant chemoradiotherapy (NCRT) on early-stage (cT1b-cT2N0M0) esophageal cancer (ESCA) and construct a prognostic nomogram for these patients.

**Methods:**

We extracted the clinical data about patients diagnosed with early-stage esophageal cancer from the 2004–2015 period of the Surveillance, Epidemiology, and End Results (SEER) database. We applied the independent risk factors affecting the prognosis of patients with early-stage esophageal cancer obtained after screening by univariate and multifactorial COX regression analyses to establish the nomogram and performed model calibration using bootstrapping resamples. The optimal cut-off point for continuous variables is determined by applying X-tile software. After balancing the confounding factors by propensity score matching (PSM) and inverse probability of treatment weighting (IPTW) method, Kaplan-Meier(K-M) curve, and log-rank test were applied to evaluate the prognostic impact of NCRT on early-stage ESCA patients.

**Results:**

Among patients who met the inclusion criteria, patients in the NCRT plus esophagectomy (ES) group had a poorer prognosis for overall survival (OS) and esophageal cancer-specific survival (ECSS) than patients in the ES alone group (*p* < 0.05), especially in patients who survived longer than 1 year. After PSM, patients in the NCRT + ES group had poorer ECSS than patients in the ES alone group, especially after 6 months, while OS was not significantly different between the two groups. IPTW analysis showed that, prior to 6 months patients in the NCRT + ES group had a better prognosis than patients in the ES group, regardless of OS or ECSS, whereas after 6 months, patients in the NCRT + ES group had a poorer prognosis. Based on multivariate COX analysis, we established a prognostic nomogram which showed areas under the ROC curve (AUC) for 3-, 5-, and 10-year OS 0.707, 0.712, and 0.706, respectively, with the calibration curves showing that the nomogram was well calibrated.

**Conclusions:**

Patients with early-stage ESCA (cT1b-cT2) did not benefit from NCRT, and we established a prognostic nomogram to provide clinical decision aid for the treatment of patients with early-stage ESCA.

## 1. Introduction

Technological advances in electronic endoscopy have made it easier to detect an increasing number of early-stage esophageal cancers (ESCA) during clinical work-ups for esophageal lesion screening, which will reduce the overall mortality rate of patients with esophageal cancer (1). According to an epidemiological survey, from 1998 to 2009, patients with early-stage esophageal cancer accounted for approximately 22% of all patients with esophageal cancer (2). Unfortunately, thoracic doctors have not yet reached a consensus on the best treatment strategy for early esophageal cancer patients (staged cT1b-cT2N0M0). For a long time, thoracic doctors have regarded ES as a standard treatment method for early-stage esophageal cancer in clinical work. However, patients with stage T1b esophageal cancer have the possibility of lymph node invasion due to invasion of the submucosa, and the rate of lymph node metastasis is higher in highly differentiated esophageal cancer tumors than in those with low differentiation (3, 4). Because stage T2 esophageal cancer has also invaded the submucosal lymphatic system and is also at high risk for lymph node metastasis (5–7), some clinicians recommend neoadjuvant therapy for patients with cT2 followed by surgical treatment (8, 9). However, regarding the efficacy of neoadjuvant therapy, many previous research results show different results, and there was even obvious controversy (10, 11).

On the other hand, for cT1b-cT2 stage patients, guidelines have recommended different treatment strategies. According to the Guide of the Chinese Society of Clinical Oncology (CSCO), esophageal cancer patients staged at cT1b-cT2 should undergo esophagectomy directly. However, the National Comprehensive Cancer Network (NCCN) have recommended patients should receive preoperative chemoradiotherapy or neoadjuvant chemoradiotherapy when the lesions were esophageal tubal division and high risk (LVI, ≥3 cm, poorly differentiated). Therefore, it is necessary to explore the differences in treatment options for early-stage esophageal cancer (stage cT1b-cT2) to guide clinicians in their treatment decisions. To our best knowledge, there is no comparison between NCRT + ES and ES for early-stage esophageal cancer patients (cT1b-cT2N0M0).

Based on these controversial treatment strategies, we carried out a retrospective study of the data of patients with esophageal malignant tumors extracted from the SEER database to compare early-staged(cT1b-cT2N0M0) esophageal cancer patients' long-term survival outcomes treated by two different strategies (NCRT + ES and ES) and analyze related risks factor. We also analyzed the variables affecting patient survival and created a nomogram with good predictive efficiency to guide decisions on clinical treatment modalities for patients.

## 2. Materials and methods

### 2.1. Study patients

We extracted the data information of early-staged ESCA patients from the 2004–2015 period from the SEER database and completed the retrospective research on these data. The SEER database, which covers approximately 30% of the U.S. national population, consists of data from 18 cancer registries (12, 13). We set esophagus as the “Site and morphology. Site recode ICD-O-3/WHO 2008 “ to identify the patients in the “selected” column. To restrict the cohort to patients with cT1b-cT2N0M0 tumors, we manually recoded the tumor-node-metastasis (TNM) stage using SEER variables or extracted directly from the database. TNM stages were determined by the sixth and seventh editions of the American Joint Committee on Cancer (AJCC) staging system. Patients whose information data were not complete such as treatment or unknown living conditions were excluded. In addition to patient demographics (age; sex; race; marital status; cause of death; survival months), cancer characteristics such as the total number of *in situ*/malignant tumors; tumor differentiation grade; histological type; tumor primary site and tumor size were extracted, and patients perform specific groups due to different baseline information and cancer characteristics including tumor differentiation grade (well-differentiated; moderately differentiated; poorly differentiated; undifferentiated, anaplastic; unknown); race (white, black, other/unknown)and histological type (adenocarcinoma, squamous cell carcinoma, other).

### 2.2. Group analysis

We also extracted a total of 737 patients who completed esophagectomy to assess the prognostic impact of NCRT, of whom 213 patients completed NCRT and 524 patients underwent ES alone. A treatment modality-based grouping was conducted for patients: neoadjuvant chemoradiotherapy followed by NCRT + ES group and only ES group. We identified surgical therapy as various forms of esophageal resection in surgical treatment, such as partial or total esophagectomy, partial or total esophagectomy with laryngectomy and/or gastrectomy and esophagectomy, with or without pharynx and laryngectomy, and these surgery methods are encoded in the SEER database 30–90. In subgroup analysis, patients were divided into high- and low-risk groups according to tumor size and tumor differentiation.

### 2.3. Statistical analysis

Through univariate and multifactorial COX analyses, we identified independent risk factors that impacted patient prognosis, derived a predictive score, and created a nomogram, and then performed model calibration.

In the subgroup analyses of treatment groups, propensity score matching (PSM) can assist to achieve the balance of the collaborative variables. Based on the logit scale, the patient propensity scores (PS) for both groups were 1:1 matched based on patients' characteristics including age, sex, race, primary tumor site, tumor differentiation grade, histological type, treatment, tumor size, marital status, and T-stage. Chi-square tests were performed after PSM to test for significant differences in categorical clinical characteristics.

We generated the survival curve using Kaplan–Meier. NCRT + ES were compared with esophagectomy alone using a log-rank test in subgroups. All statistical analyses and graph plotting were performed using R (4.1.2). All analyses were two-sided, with *p* < 0.05 considered statistically significant.

## 3. Results

### 3.1. Patient demographics

**Figure 1:**
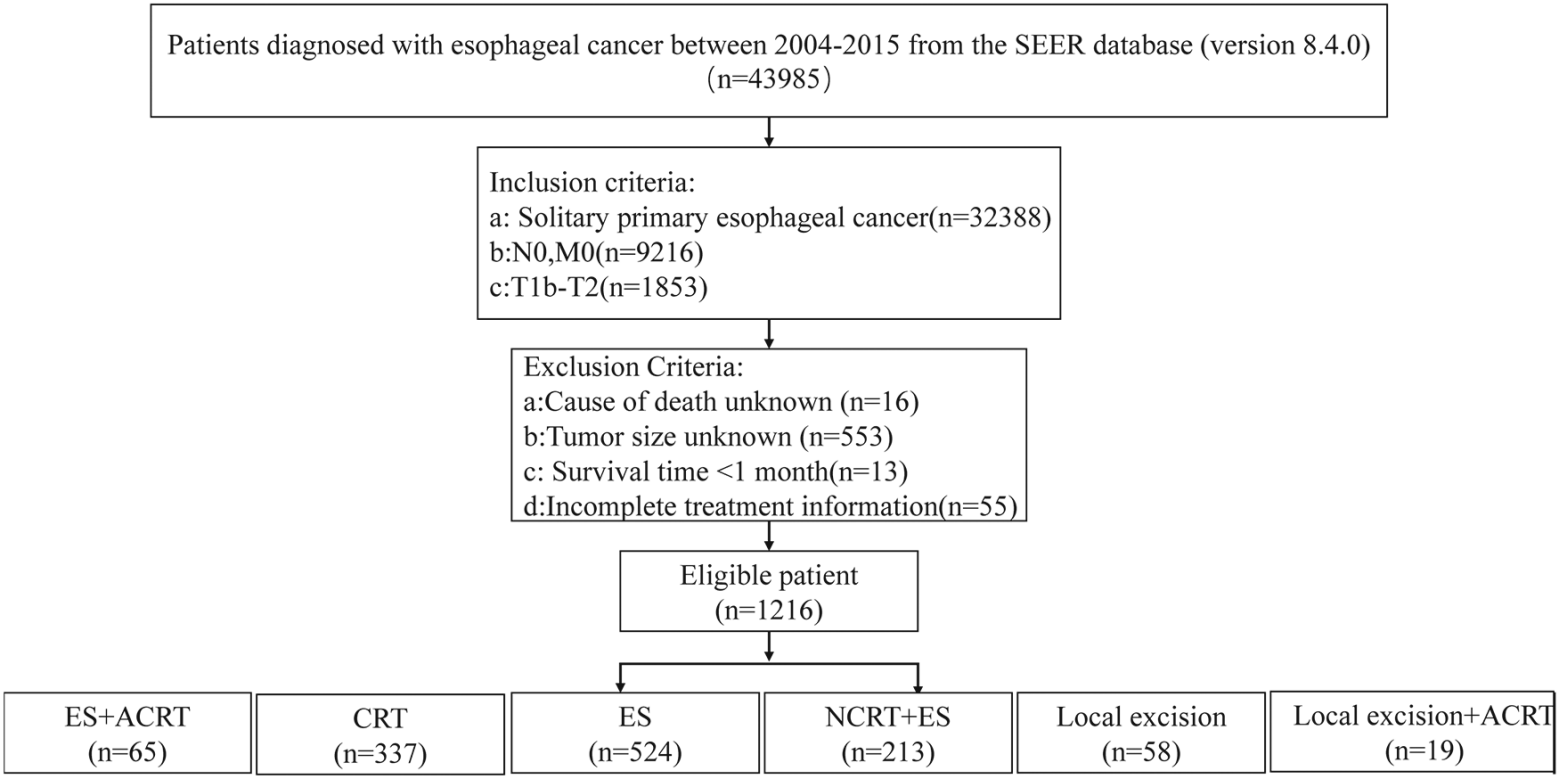
The flowchart of the data filtering process. SEER, Surveilance, Epidemiology, and End Results; NCRT + ES, neoadjuvant chemoradiotherapy plus esophagectomy; ES, esophagectomy; CRT, chemoradiotherapy; ACRT, adjuvant chemoradiotherapy.

A total of 1,216 patients met the inclusion criteria, and the screening process is shown in Figure 1, and the clinical baseline information of these patients is shown in Table 1. The majority of patients are middle-aged and elderly (45–75 years, 77.1%) men (79.4%), predominantly Caucasian (89.3%), with the majority of adenocarcinoma (68.8%) of the lower esophagus (69.5%), and more patients undergo the treatment modality of esophagectomy alone (43.1%). The baseline information on patients in the subgroup analysis assessing the impact of neoadjuvant chemoradiotherapy is shown in Table 2. More black people with stage T2 and unclear tumor differentiation were treated with NCRT + ES than other ethnic groups.

**Table 1:** Demographics and clinical characteristics of patients from the SEER database.

Characteristic	Levels	Overall
*n*		1216
**Age, *n* (%)**		
	<45 years	18 (1.5%)
	45–64	507 (41.7%)
	65–75	431 (35.4%)
	>75	260 (21.4%)
**Race, *n* (%)**		
	White	1086 (89.3%)
	Black	67 (5.5%)
	Other	63 (5.2%)
**Sex, *n* (%)**		
	Male	966 (79.4%)
	Female	250 (20.6%)
**Primary site, *n* (%)**		
	Upper	48 (3.9%)
	Middle	203 (16.7%)
	Lower	845 (69.5%)
	Overlapping	120 (9.9%)
**Treatment, *n* (%)**		
	ES	524 (43.1%)
	ES+ACRT	65 (5.3%)
	NCRT+ES	213 (17.5%)
	CRT	337 (27.7%)
	Local excision	58 (4.8%)
	Local excision +ACRT	19 (1.6%)
**Tumor size, *n* (%)**		
	<1.8 cm	353 (29%)
	≥1.8 cm	863 (71%)
**Marital status, *n* (%)**		
	Married	756 (62.2%)
	Unmarried	460 (37.8%)
**Grade, *n* (%)**		
	Grade I	134 (11%)
	Grade II	565 (46.5%)
	Grade III	378 (31.1%)
	Grade IV	20 (1.6%)
	Unknown	119 (9.8%)
**Histologic type, *n* (%)**		
	ESCC	315 (25.9%)
	EAC	836 (68.8%)
	Other	65 (5.3%)
**Regional LN scope, *n* (%)**		
	None	472 (38.8%)
	1–3	87 (7.2%)
	≥4	657 (54%)
**Stage T, *n* (%)**		
	T1b	542 (44.6%)
	T2	674 (55.4%)

**Table 2:** Baseline characteristics before and after PSM analysis.

Characteristic	Before matching			After matching		
	ES	NCRT+ES	*p*	ES	NCRT + ES	*p*
*N*	524	213		142	142	
**Age, *n*** (**%)**			0.001*			0.345*
<45	9 (1.2%)	4 (0.5%)		6 (2.1%)	2 (0.7%)	
45–64	239 (32.4%)	124 (16.8%)		61 (21.5%)	70 (24.6%)	
65–75	193 (26.2%)	70 (9.5%)		56 (19.7%)	56 (19.7%)	
>75	83 (11.3%)	15 (2%)		19 (6.7%)	14 (4.9%)	
**Race, *n*** (**%)**			< 0.001			0.033
White	480 (65.1%)	186 (25.2%)		133 (46.8%)	122 (43%)	
Black	13 (1.8%)	20 (2.7%)		3 (1.1%)	13 (4.6%)	
Other	31 (4.2%)	7 (0.9%)		6 (2.1%)	7 (2.5%)	
**Sex, *n*** (**%)**			0.691			0.884
Male	429 (58.2%)	171 (23.2%)		111 (39.1%)	113 (39.8%)	
Female	95 (12.9%)	42 (5.7%)		31 (10.9%)	29 (10.2%)	
**Primary_site, *n*** (**%)**			0.095*			0.423*
Upper	12 (1.6%)	1 (0.1%)		0 (0%)	1 (0.4%)	
Middle	81 (11%)	30 (4.1%)		22 (7.7%)	21 (7.4%)	
Lower	379 (51.4%)	169 (22.9%)		106 (37.3%)	112 (39.4%)	
Overlapping	52 (7.1%)	13 (1.8%)		14 (4.9%)	8 (2.8%)	
**Tumor_size, *n*** (**%)**			< 0.001			0.895
≤1.8 cm	198 (26.9%)	49 (6.6%)		39 (13.7%)	41 (14.4%)	
>1.8 cm	326 (44.2%)	164 (22.3%)		103 (36.3%)	101 (35.6%)	
**Marital_status, *n*** (**%)**			0.219			1.000
Married	335 (45.5%)	147 (19.9%)		92 (32.4%)	91 (32%)	
Unmarried	189 (25.6%)	66 (9%)		50 (17.6%)	51 (18%)	
**Grade, *n*** (**%)**			<0.001			0.213
Grade I	81 (11%)	19 (2.6%)		10 (3.5%)	16 (5.6%)	
Grade II	263 (35.7%)	92 (12.5%)		76 (26.8%)	65 (22.9%)	
Grade II	153 (20.8%)	76 (10.3%)		44 (15.5%)	50 (17.6%)	
Grade IV	13 (1.8%)	0 (0%)		3 (1.1%)	0 (0%)	
Unknown	14 (1.9%)	26 (3.5%)		9 (3.2%)	11 (3.9%)	
**Histologic_type, *n*** (**%)**			0.084			0.615
ESCC	95 (12.9%)	54 (7.3%)		30 (10.6%)	37 (13%)	
EAC	407 (55.2%)	150 (20.4%)		105 (37%)	98 (34.5%)	
Other	22 (3%)	9 (1.2%)		7 (2.5%)	7 (2.5%)	
**Stage_T, *n* (%)**			<0.001			1.000
T1b	383 (52%)	36 (4.9%)		36 (12.7%)	36 (12.7%)	
T2	141 (19.1%)	177 (24%)		106 (37.3%)	106 (37.3%)	

* Indicates that the *p*-value is the result of the Fisher.test to distinguish it from other *p*-values of the Chisq.test test.

### 3.2. Construction of the nomogram

**Figure 2:**
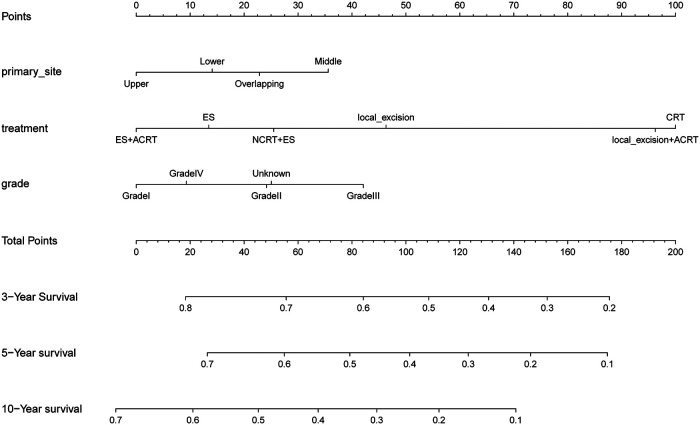
The nomogram of predicting 1-, 3-, and 5-year overall survival rates for cT1b-cT2 stage patients.

The results of the univariate Cox regression analysis in the training cohort show that seven variables including age, primary site, treatment, tumor size, grade, histologic type, and T stage were potential risk factors of OS shown in Supplementary Table S1. We performed a multifactorial COX regression analysis of these seven risk factors to screen out 3 independent prognostic risk factors shown in Supplementary Table S2. Next, we completed the derivation of predictive score to construct nomograms of these indicators (Figure 2). The nomogram predicted overall survival at 3, 5, and 10 years for early-stage esophageal cancer. Based on the total scores obtained from the scores corresponding to the variables, the factors that had the greatest impact on patient prognosis were age, treatment modality, and degree of tumor differentiation.

### 3.3. Model calibration

**Figure 3:**
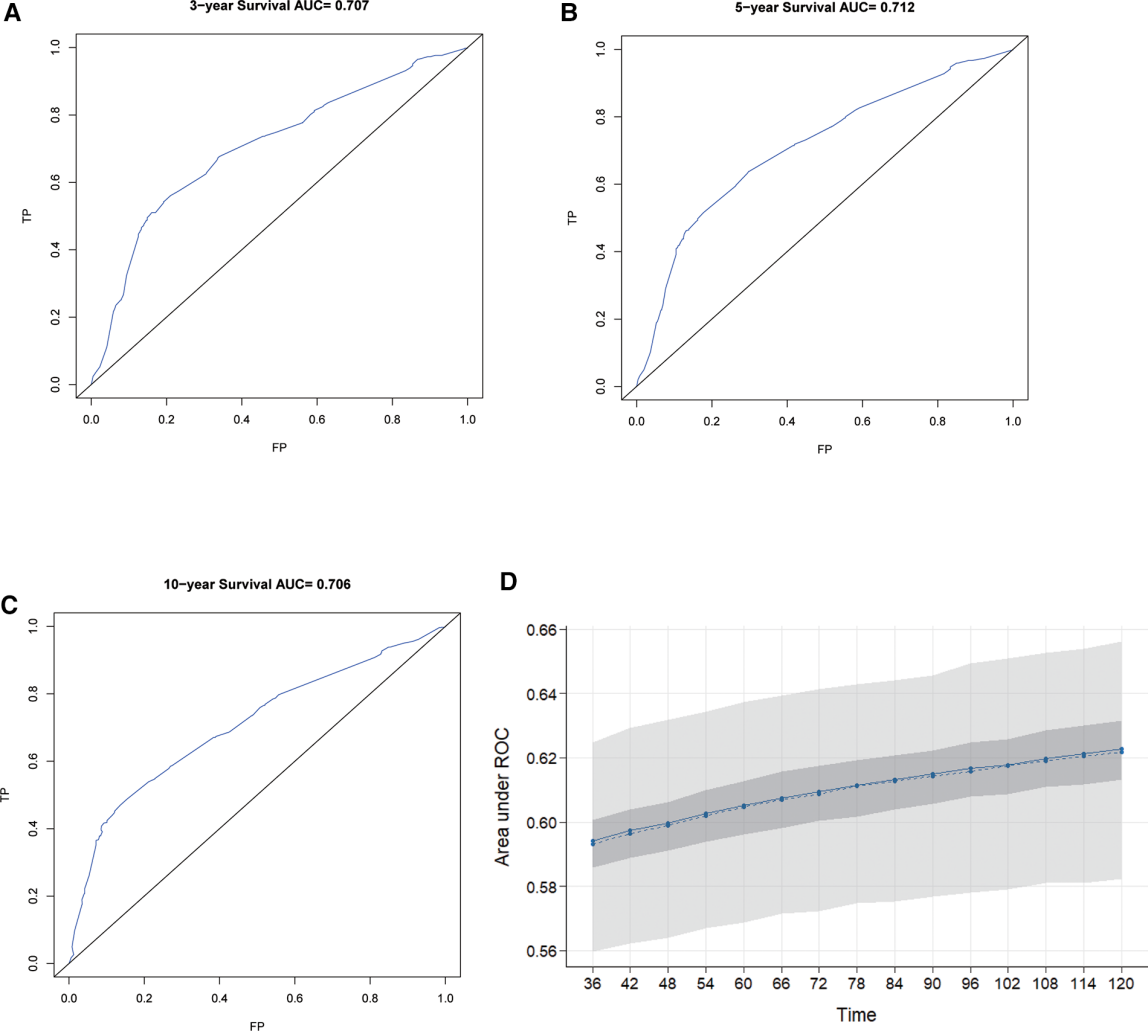
ROC curves for 3-year, 5-year, and 10-year OS predicted by the nomogram (**A–C**) and model calibration (**D**).

**Figure 4:**
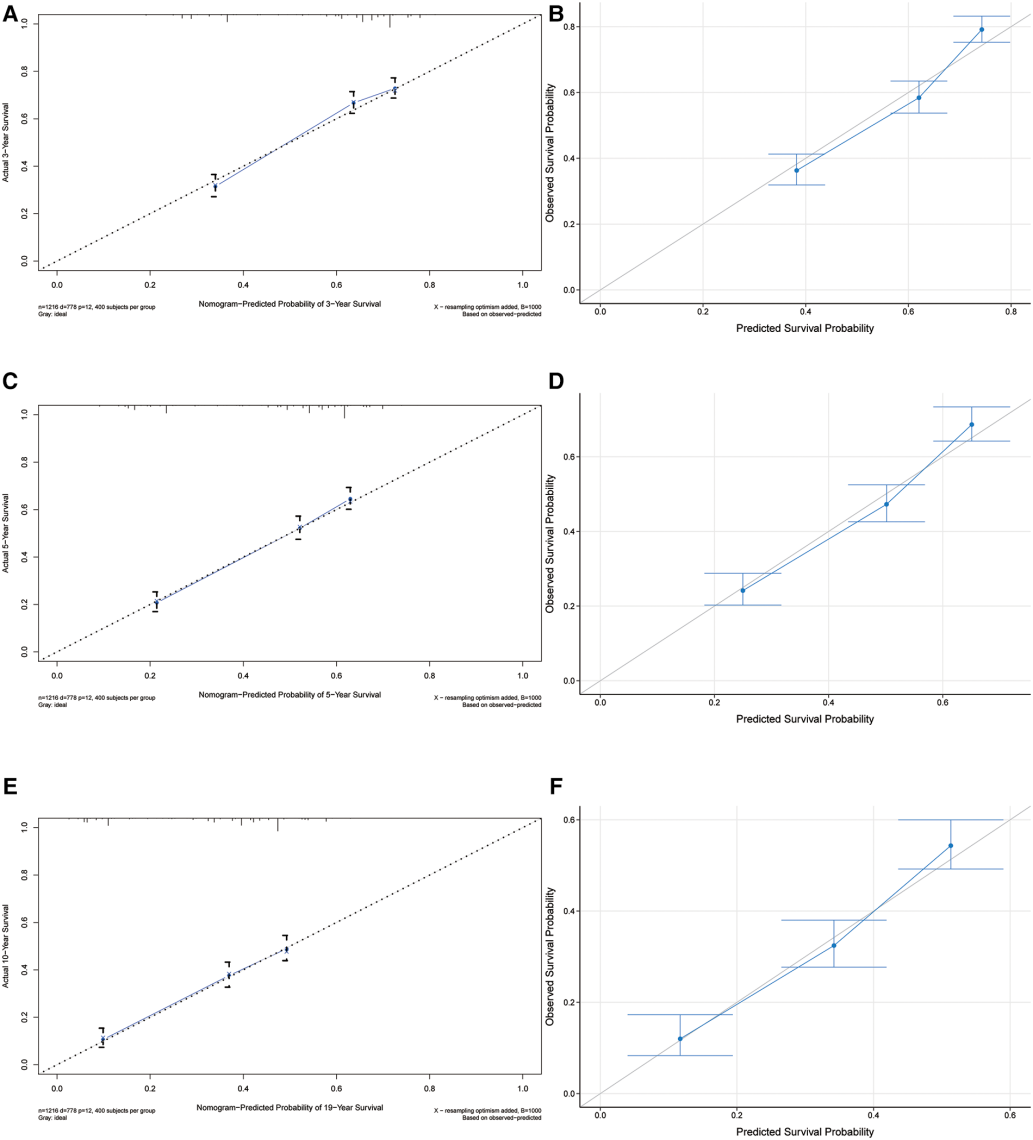
Calibrate curves for 3-year, 5-year, and 10-year OS predicted by the nomogram (**A,C,E**) and model callibration (**B,D,F**).

**Figure 5:**
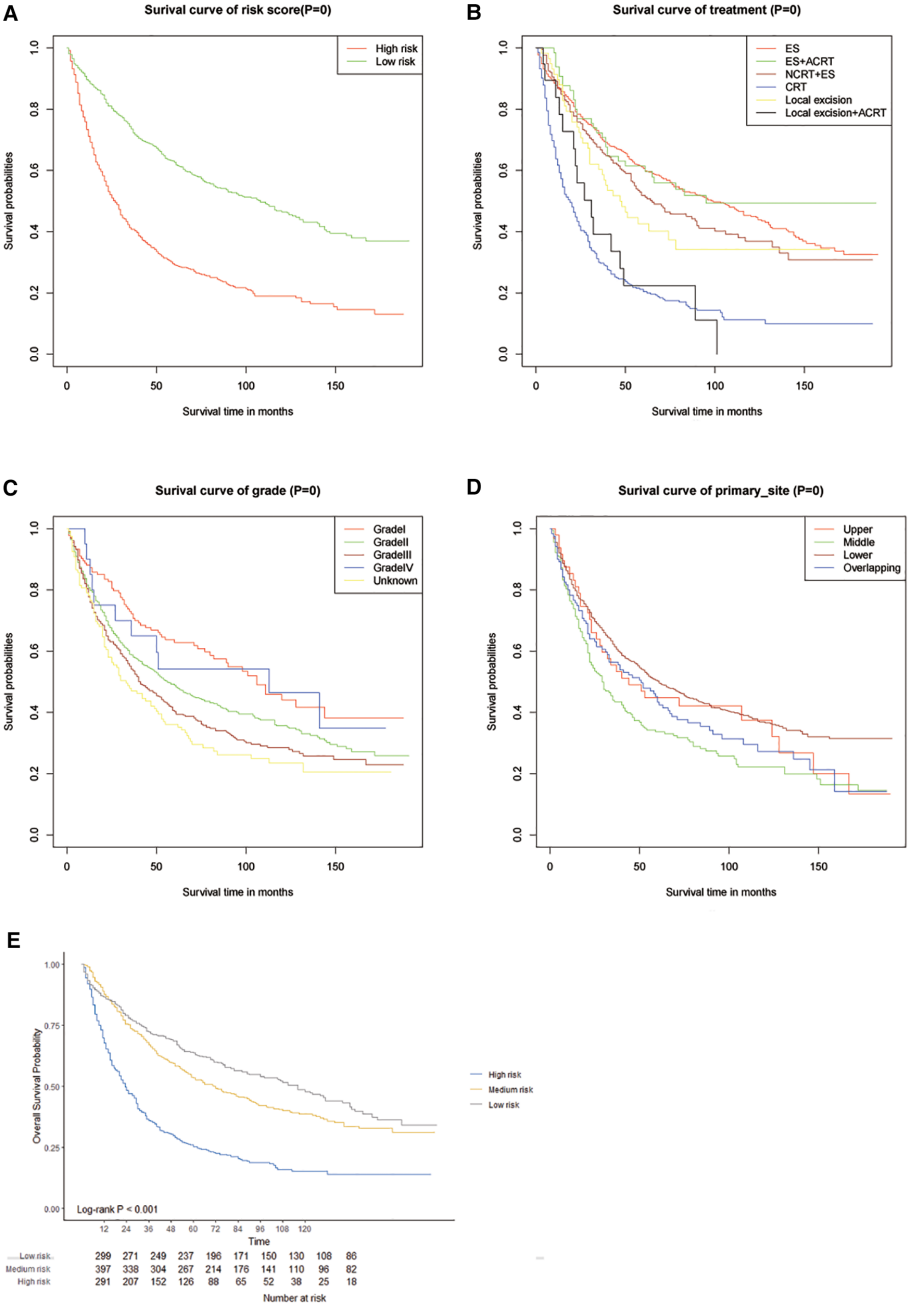
Kaplanmeier survival curves in different score cohorts contibuted by the nomogram (**A–D**) and calliberated (**E**).

The C-index of our developed prediction model was 0.65 [95% confidence interval (CI): 0.63–0.67], and the AUC values of the ROC tests for 3, 5, and 10 years were 0.707, 0.712, and 0.706, and were calibrated using bootstrapping resamples shown in Figure 3. Calibration curves showed agreement between predicted and observed 3, 5, and 10-year survival probabilities (Figure 4). Finally, we performed a Kaplan-Meier survival analysis on the patients, and the results are shown in Figure 5.

### 3.4. Subgroup analysis

**Figure 6:**
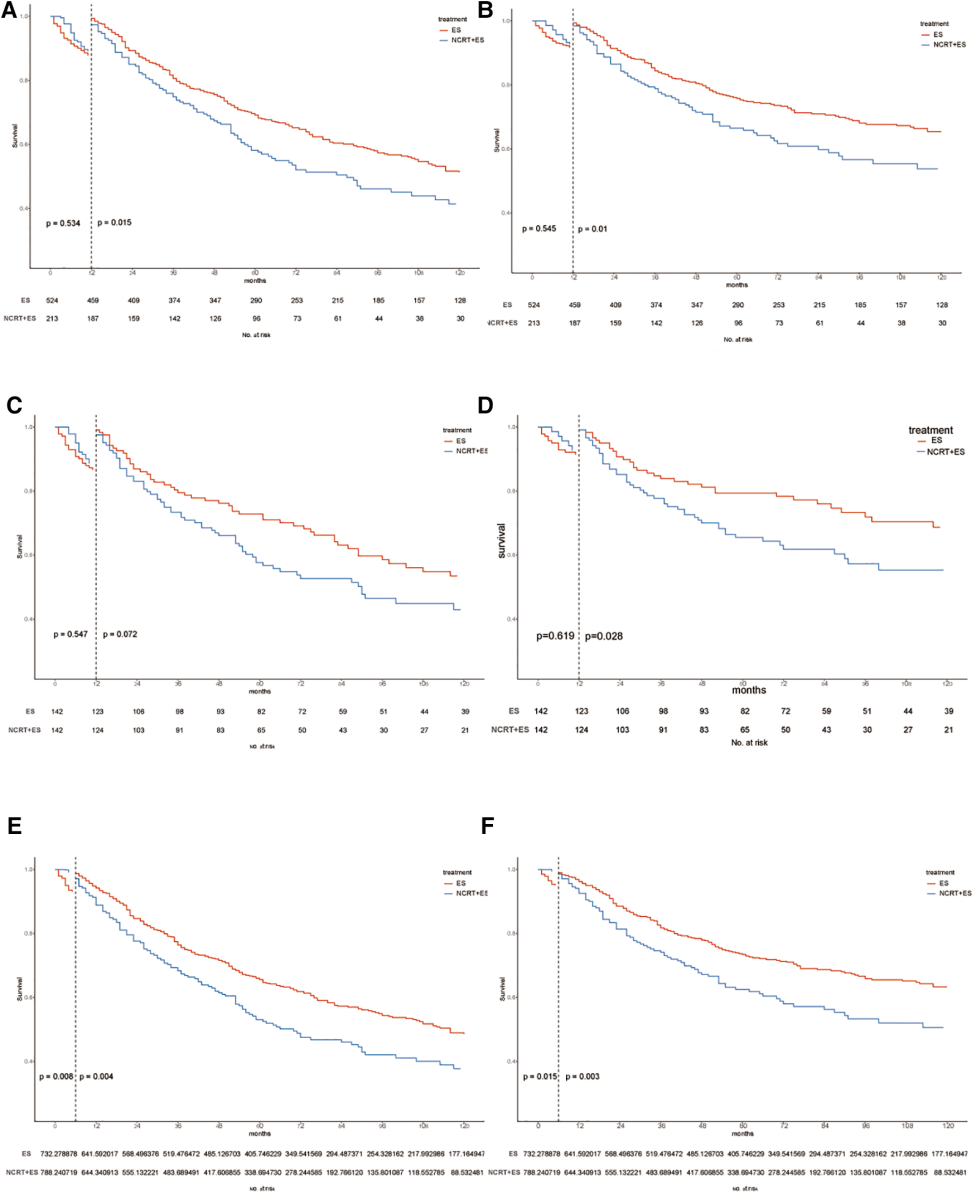
Kaplan–Maier curves of OS and ECSS estimates for cT1b-cT2 stage esophagus cancer with or without neoadjuvant chemoradiotherapy in three cohorts. OS and ECSS of pre-match cohort (**A,B**), post-match cohort (**C,D**) and IPTW cohort (**E,F**).

In Figure 6, Kaplan–Meier survival curves are shown for the OS and ECSS curves in three cohorts including unmatched, PSM-matched, and inverse probability of treatment weighting (IPTW) patient cohorts. In both the unmatched and IPTW-weighted cohorts, patients with early-stage ESCA treated with NCRT combined with ES had worse overall survival and esophageal cancer-specific survival than those who undergo direct ES after 6 months (*p* < 0.05). And in the PSM-matched cohort, patients in the NCRT + ES group also had poorer ECSS than those in the ES group after 6 months (*p* < 0.05).

## 4. Discussion

So far, this study is the first study that integrates the analysis of prognostic factors in patients with early-stage (cT1b-cT2) ESCA administered multiple treatment modalities. In this study, the primary tumor site, treatment modality, and the degree of tumor differentiation were found to be independent prognostic risk factors. On this basis, we established a prognostic nomogram for early ESCA with some accuracy and high calibration consistency. Although predictive models of esophageal cancer prognosis were developed in the nomogram of other groups. However, studies on the prognostic impact of neoadjuvant chemoradiotherapy for early esophageal cancer without lymph node metastasis and the establishment of nomograms have not been reported (14–16). The present study therefore also evaluated the adverse effects of NCRT on the prognosis of patients with early ESCA.

According to the clinical staging of the AJCC staging system in the eighth edition, neither tumor location nor the pathological grade of the tumor was a staging factor for patients with ESCA. Interestingly, in our study, we found that both tumor location and pathological grade of tumor differentiation were independent risk factors in patients with the cT1b-cT2 stage ESCA.

There is no consensus on the optimal treatment of patients with cT1b-cT2N0 stage ESCA. In our study, we therefore investigated whether NCRT had a potentially protective effect on patients with cT1b-cT2 stage ESCC. Our results showed that patients with cT1b-cT2 stage esophageal cancer couldn't benefit from neoadjuvant chemoradiotherapy. In contrast, patients who received NCRT + ES after a 6-month follow up period had poorer OS and ESCC than those who received ES alone. This may be related to the side effects of chemoradiotherapy. Previous studies demonstrated that NCRT caused toxicity and adverse effects on quality of life and higher postoperative mortality (17–19). Although some studies have reported that neoadjuvant chemotherapy is beneficial to the prognosis of patients with stage I or II, resectable esophageal cancer (20, 21). However, related studies have found no significant effect of neoadjuvant therapy on survival of patients with esophageal cancer (22). Furthermore, as our study results in 3 cohorts, NCRT + ES was associated with poorer long-term prognosis of OS and CSS than ES alone therapy, which is consistent with the previous results (17). It is therefore recommended that cT1b-cT2N0 esophageal cancer patients undergo an esophagectomy alone as their primary treatment.

Previous research showed that neoadjuvant therapy could increase side effects such as pneumonia, arrhythmia, and postoperative deaths (23, 24). A neoadjuvant chemotherapy followed by surgery in cT1b-cT2 patients led to poor ECSS, perhaps because of these adverse effects. Jeremiah T Martin et.al reported that the treatment strategy of radiation plus esophagectomy did not improve outcomes for T2N0 esophageal cancer patients compared to esophagectomy alone in the SEER database (25), a finding consistent with the results of the present study. Thus, a more comprehensive study of the treatment of cT1b-cT2 ESCA is needed to determine the optimal treatment for these patients.

Clinical staging of esophageal cancer plays an important role in treatment decisions. The determination of lymph node status is a particularly important aspect of clinical staging. Patients with cT1 stage ESCA were found to be at risk of lymph node metastasis (3, 4). The association of preoperative clinical staging with the risk of lymph node metastasis was found to be somewhat inaccurate for patients with stage cT2 ESCA (5, 6). However, the increased use of endoscopic physical examination and further developments in treatment technology have led to increased rates of detection of early ESCA (26, 27). This, in turn, may result in increased accuracy of clinical staging. Prospective studies including large numbers of patients are required to assess the accuracy of clinical staging of early-stage ESCA, thereby optimizing individualized treatment.

Several advantages of this study include its large sample size and complete long-term follow-up information, which allowed us to see the effects of different treatment methods on patients with esophageal cancer. This study has, of course, some limitations. First of all, as a retrospective and non-randomized study, this study has some inevitable bias. In addition to the PSM, we use the IPTW analyses for confounder balance. Secondly, the accuracy of clinical stage cannot be evaluated without the pathological stage being confirmed and a large-size prospective study is expected to provide detailed information about long-term prognoses of cT1b-cT2N0 stage esophageal cancer patients treated with different methods. Thirdly, detailed radiotherapy information, such as the dose of radiotherapy, the number of chemotherapy sessions, and the chemotherapy regimen, is not available in the database. In addition, the performance of the predictive nomogram requires a validation in a different patient cohort.

In conclusion, the present study found that tumor location, degree of tumor differentiation, and treatment modality were independent prognostic risk factors for patients with early-stage (cT1b-cT2) ESCA, and NCRT was an unfavorable factor for patient prognosis.

## Data Availability

The original contributions presented in the study are included in the article/**Supplementary Material**, further inquiries can be directed to the corresponding author.
