# Supplementary material for: Influence of adverse effects of neoadjuvant chemoradiotherapy on the prognosis of patients with early-stage esophageal cancer (cT1b-cT2N0M0) based on the SEER database
Source: Front Surg. 2023 Apr 17;10:1131385. doi: 10.3389/fsurg.2023.1131385 (PMC10153569; doi:10.3389/fsurg.2023.1131385)
Supplement: Supplementary file 1 [file Table1.docx]

| Table S1 Univariate analysis of overall survival. | | | |
| --- | --- | --- | --- |
| Variable | Univariate analysis | | |
|  | exp(coef) | 95% CI | P value |
| Age | 1.58274 | 1.447-1.731 | <2e-16 |
| Race | 0.98266 | 0.8523-1.133 | 0.81 |
| Sex | 1.0224 | 0.8592-1.217 | 0.803 |
| Primary site | 0.8814 | 0.7889-0.9848 | 0.0257 |
| Treatment | 1.30012 | 1.24-1.364 | <2e-16 |
| Tumor size | 1.3306 | 1.134-1.561 | 0.000462 |
| Marital status | 1.11348 | 0.9638-1.286 | 0.144 |
| Grade | 1.15276 | 1.082-1.229 | 1.22E-05 |
| Histologic type | 0.75753 | 0.6575-0.8728 | 0.000122 |
| Stage T | 1.66228 | 1.438-1.921 | 6.01E-12 |
