# Supplementary material for: Influence of adverse effects of neoadjuvant chemoradiotherapy on the prognosis of patients with early-stage esophageal cancer (cT1b-cT2N0M0) based on the SEER database
Source: Front Surg. 2023 Apr 17;10:1131385. doi: 10.3389/fsurg.2023.1131385 (PMC10153569; doi:10.3389/fsurg.2023.1131385)
Supplement: Supplementary file 2 [file Table2.docx]

| Table S2 Multivariate Cox analysis of overall survival. | | | |
| --- | --- | --- | --- |
| Variable | Multivariate analysis | | |
|  | HR (95% CI) | | P value |
| Age |  |  |  |
| <45 |  |  |  |
| 45-64 | 1.07014 | 0.5279-2.169 | 0.85085 |
| 65-75 | 1.57841 | 0.7788-3.199 | 0.20537 |
| >75 | 1.96496 | 0.9609-4.018 | 0.06421 |
| Treatment |  |  |  |
| ES |  |  |  |
| ACRT | 0.78979 | 0.5401-1.155 | 0.22366 |
| NCRT+ES | 1.10331 | 0.8682-1.402 | 0.42144 |
| CRT | 2.3147 | 1.8663-2.871 | **2.19E-14** |
| excision | 1.33344 | 0.9338-1.904 | 0.11334 |
| excision+ACRT | 2.37709 | 1.4212-3.976 | **0.00097** |
| Grade |  |  |  |
| GradeII | 1.25859 | 0.9596-1.651 | 0.09647 |
| GradeIII | 1.52225 | 1.1505-2.014 | **0.00326** |
| GradeIV | 0.94282 | 0.4933-1.802 | 0.85857 |
| Unknown | 1.31451 | 0.9431-1.832 | 0.10648 |
| Stage |  |  |  |
| T1b |  |  |  |
| T2 | 1.18099 | 0.9831-1.419 | 0.0754 |
| Tumor primary site |  |  |  |
| Upper |  |  |  |
| Middle | 1.5054 | 1.0201-2.222 | **0.03938** |
| Lower | 1.23117 | 0.8353-1.815 | 0.29338 |
| Overlapping | 1.26545 | 0.8324-1.924 | 0.2706 |
| Tumor size |  |  |  |
| <1.8cm |  |  |  |
| ≥1.8cm | 1.0959 | 0.925-1.298 | 0.28981 |
| Histologic type |  |  |  |
| ESCC |  |  |  |
| EAC | 0.94588 | 0.7754-1.154 | 0.58325 |
| Other | 1.24977 | 0.8823-1.77 | 0.20939 |
